# A nationwide cohort study on the association between intensive care treatments and mental distress linked psychiatric disorders

**DOI:** 10.1038/s41598-024-55102-9

**Published:** 2024-02-24

**Authors:** Rasmus Mossberg, Björn Ahlström, Miklos Lipcsey

**Affiliations:** 1 Anesthesiology and Intensive Care, Department of Surgical Sciences, Uppsala University, Uppsala, Sweden; 2 Region Värmland, Center for Clinical Research Värmland, Centralsjukhuset Karlstad, Rosenborgsgatan 9, 65230 Karlstad, Sweden; 3 Healthcare Region Dalarna, Center for Clinical Research Dalarna, Falu Lasarett, Nissers väg 3, 79182 Falun, Sweden; 4 Hedenstierna Laboratory, Department of Surgical Sciences, Uppsala University, 75185 Uppsala, Sweden

**Keywords:** Mental disorders, Critical illness, Intensive care units, Epidemiology, Risk factors, Epidemiology, Outcomes research, Risk factors, Renal replacement therapy

## Abstract

Given the psychic strain patients experience in the intensive care unit (ICU), a potential risk of mental disorders has been suggested. However, the effects of intensive care treatment per se are unknown. We investigated whether the level of intensive care treatments is an independent risk factor for developing long-term mental disorders after intensive care. In a national cohort of adult ICU patients we combined data on diagnoses, treatment, and causes of death. We defined extensive ICU treatment as being treated with invasive ventilation for > 24 h, continuous renal replacement therapy, or both. The primary outcome was incident mental disorder 1 year after ICU admission. Extensive ICU treatment was found to be associated with a decreased risk of developing a mental disorder ≥ 1 year after ICU admission (HR 0.90, 95% CI 0.82–0.99, *p* = 0.04), and increasing severity of acute illness (HR 1.18, 95% CI 1.06–1.32, *p* < 0.001) were associated with an increased risk of mental disorders. Because death acted as a competing risk for mental illness, mortality might help explain the apparent protective effect of extensive ICU care.

Trial registration Clinical Trials Registry (Identification number NCT05137977). Registered 16 November 2021. As a registry trial the patients were already included at the trial registration i.e. it was retrospectively registered.

## Introduction

The focus on intensive care unit (ICU) outcomes has increasingly turned from mortality as a sole variable to including post-ICU stay morbidity and quality of life^1,2^. Patients with a critical illness are exposed to various factors causing mental distress, such as pain, fear of death, altered level of conscientiousness, disturbed day-night cycle, respiratory distress, and delirium. Subsequent ICU care can add to this burden by exposing patients to therapy (e.g., drugs that might trigger an altered perception of reality, inadequate ventilatory support, and frightening situations related to themselves or other patients. Consequently, it has been suggested that severe illness or intensive care experience might trigger mental disorders, including post-traumatic stress disorder (PTSD)^3^, post-ICU depressive states, or anxiety syndromes^4^. PTSD is a syndrome characterized by exposure to a traumatic event that has been or is perceived as life-threatening. It has been theorized it would especially affect ICU patients^3,5^.

Additionally, depressive states and anxiety syndromes have been associated with severe somatic illness during ICU admission^6–10^. Few studies have investigated the effects of different treatments and the extent of the impact of ICU intervention on later mental disorders^11–15^. These studies vary considerably in study design, size, and report conflicting results. Either they reported cohorts of ICU patients regardless of treatment, or ICU patients were compared to hospital-admitted patients, rendering the effect of the ICU treatments largely unknown.

We hypothesized that more extensive ICU care (i.e., organ support) would increase the rate of long-term mental disorders after ICU admission. We further hypothesized that earlier known risk factors for mental disorders would also increase the risk of mental disorders in this setting. Thus, we aimed to investigate how intensive care treatment contributes to the psychiatric sequelae after ICU. Accordingly, we evaluated the prevalence of mental disorders after intensive care in a nationwide cohort of ICU patients in Sweden. Using personal identification numbers (PINs), we linked a national ICU quality registry and governmental registries on all inpatient care and deaths to perform a cohort study.

## Methods

This study was approved by the Regional Ethics Committee of Uppsala (approval No. 2016/421) and as a registry-based study individual consent was waived. It was registered a priori with the Clinical Trials Registry (Identification number NCT05137977, registered November 16, 2021). Reporting complies with the STROBE (STrengthening the Reporting of OBservational studies in Epidemiology) statement^16^ and the declaration of Helsinki and its subsequent revisions were followed.

### Cohort

We included all patients aged ≥ 18 years and who were admitted to an ICU and registered in the Swedish intensive care registry (SIR)^17^ between 2005 and 2015. Patients with prior diagnosis codes corresponding to mental disorders as described for the outcome, having an ICU stay of < 24 h, and those that died within 1 year of intensive care were excluded. After exclusion, we divided patients into two groups based on the extent of ICU treatment: Extensive and non-extensive ICU treatment. Extensive ICU treatment was defined as either receiving invasive mechanical ventilation (IMV) for > 24 h, receiving any continuous renal replacement therapy (CRRT), or both. After exclusion and without exposure to extensive ICU care, the remaining patients were included in the non-extensive ICU treatment group.

### Data sources

SIR, the National patient register (NPR)^18^, and the Cause of Death Register (CDR)^19^ were used. SIR is a national intensive care registry to which all ICU admissions and discharges are reported. The registry covered 56% of Sweden’s ICUs in 2005, 92% in 2012, and 94% in 2016. The NPR includes data from all inpatient hospital visits while CDR registers contain all deaths of Swedish residents. The latter two registries are maintained by the Swedish National Board of Health and Welfare, where reporting is mandatory by statuary and common law.

### Data

From the SIR, we extracted discharge diagnoses according to the International Classification of Diseases, Tenth Revision (ICD 10) codes^20^, Swedish operational procedure codes^21^, data on invasive ventilation, CRRT, date of ICU admission and discharge, sex, age, and data on the severity of illness at admission. Severity of illness was initially reported as Acute Physiology, age, chronic health evaluation II (APACHE II)^22^ to the SIR and, during 2010, substituted with Simplified Acute Physiology Score 3 (SAPS3)^23^. Overlapping and adjacent ICU episodes, separated by < 24 h, were merged. Date and cause of death were extracted from the CDR; from the NPR, we extracted ICD-10 codes for all inpatient care episodes from 5 years before the ICU admission to December 31, 2016.

### Outcomes

The primary outcome—incident mental disorders—was defined as having an ICD 10-code from at least one mood (affective) disorder, reaction to severe stress, adjustment disorders, PTSD, suicide, SUD, ISH, and other anxiety disorders. The ICD-10 codes used can be found in Supplementary Table S2. We began follow-up 1 year after ICU admission to ascertain that we assessed long-term mental disorders and not short-term stress reactions—subsequently, any incident mental disorders during the first year after ICU admission were ignored.

### Statistics

We present descriptive statistics, the number of observations with percentages, means with standard deviations, and medians with interquartile ranges. Pearson’s chi-squared test and Kruskal–Wallis one-way analysis of independence were used for univariate comparisons. For the unadjusted survival analysis, a Log-rank test was calculated. We used a Cox proportional hazards regression model with incident mental disorders as the dependent variable. After a literature review, we added previous traumatic brain injury (TBI), cerebrovascular accident (CVA), cardiac arrest (CA), cardiopulmonary bypass (CPB), and extracorporeal membrane oxygenation (ECMO) as confounding variables in the model^7–10,13^. The ICD-10 and procedure codes appear in Supplementary Table S3. We defined ‘previous’ as a diagnosis reported before or within 4 weeks of ICU admission. Age and sex were added to the model with the updated Charlson comorbidity index (CCI)^24,25^ and an adjusted SAPS3. Because age and comorbidities were included in our model, points for those were subtracted from SAPS3, rendering SAPS3a. Because we assumed nonlinearity for age and SAPS3 against mental disorders, we applied restricted cubic splines to those variables, and hazard ratios (HRs) were calculated between the 25th and 75th percentile.

We performed several sensitivity analyses, all specified in Supplementary Tables S4–S16. The Cox model was repeated, including incident mental disorders from ICU admission and later. We performed the model separately on each group of ICD-10 codes from our primary outcome (i.e., mental disorders). Moreover, the extensive ICU parameter was substituted with IMV > 24 h and CRRT separated in the model. In a Cox model we also used a stricter definition of extensive ICU care requiring both CRRT and IMV > 24 h. Moreover, the model was performed on complete cases only. We also excluded TBI, CVA, CA, CPB, and ECMO from the model. Finally, a Fine-Gray analysis was performed with death as a competing event.

A two-sided *p*-value < 0.05 was considered statistically significant. Missing SAPS3 data were imputed using multiple imputations by chained equation. The resultant model outputs were pooled. Data management and descriptive statistics were performed in SPSS for windows version 26.0.0.0 (Microsoft Inc., IL, USA). Cox models, univariate statistical testing, and imputations were performed in the R software version 4.1.2 (The R foundations for statistical computing, Vienna, Austria).

## Results

**Figure 1:**
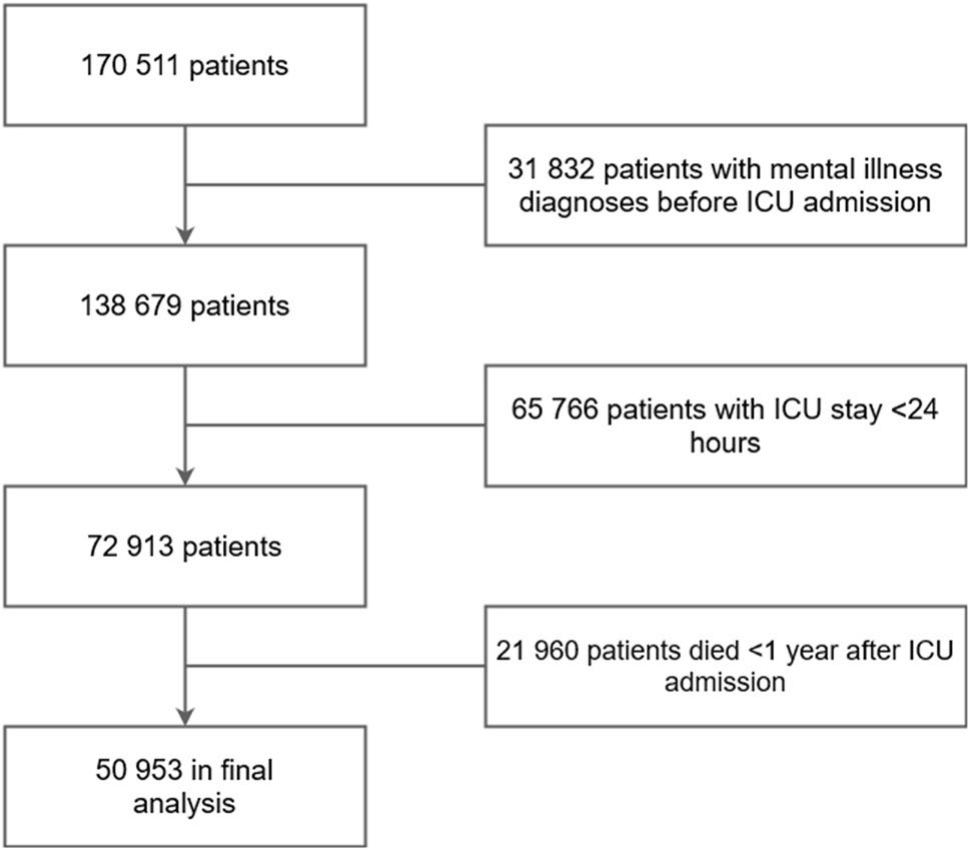
Patient selection flowchart. ICU, intensive care unit.

Of 170 511 patients, 50 953 were included in the cohort. This cohort had no previous mental disorders, had an ICU stay > 24 h, and was alive 1 year after admission (Fig. 1).

Median follow-up time was 2020 days (IQR 1321–2738). Few cases (n = 22) had a possible loss to follow-up because of emigration after ICU admission. Individuals who emigrated remained in the cohort. In the final cohort 13 062 patients (25.6%) were allocated to the extensive ICU-treatment group and 37 891 (74.4%) to the non-extensive ICU-treatment group (Table 1).

**Table 1:** Patient characteristics, stratified after intensive care level.

	Extensive ICU treatment cohort	Non-extensive ICU treatment cohort	All patients
Number of patients	13,062 (25.6)	37,891 (74.4)	50,953 (100)
Female sex***	5091 (29)	15,140 (42.6)	21,231 (41.7)
Age at ICU admission (Years)***	64 (52–72)	66 (52–75)	66 (52–74)
Surgery***	2134 (16.3)	4185 (11)	6319 (12.4)
SAPS3***	59 (50–69)	49 (41–59)	52 (42–62)
CCI***	0 (0–2)	0 (0–2)	0 (0–2)
IV***	12,405 (95)	9161 (24.2)	21,566 (42.3)
IMV (> 24 h)	12,208 (93.5)	0 (0)	12,208 (24)
IMV time (minutes)***	5911 (2780–13,795)	392 (180–888)	2115 (525–7170)
CRRT	2604 (19.9)	0 (0)	2604 (5.1)
CRRT time (minutes)	5465 (2700–11,187)	(-)	5465 (2700–11,187)
IMV (> 24 h) and CRRT	1750 (13.4)	0 (0)	1750 (3.4)
ICU LoS (hours)***	174 (92–340)	46 (32–73)	58 (37–118)
TBI***	1432 (11)	3055 (8.1)	4487 (8.8)
CVA***	2299 (17.6)	5451 (14.4)	7750 (15.2)
CA***	1442 (11)	808 (2.1)	2250 (4.4)
CPB***	1034 (7.9)	6070 (16)	7104 (13.9)
ECMO***	69 (0.5)	21 (0.1)	90 (0.2)

Variables listed in order: *CA* cardiac arrest, *CVA* cerebrovascular accident, *CCI* Charlson comorbidity index, *CRRT* continuous renal replacement therapy, *ECMO* extracorporeal membrane oxygenation, *ICU* intensive care unit, *ISH* intentional self-harm, *IV* invasive ventilation, *LoS* length of stay, *SAPS3* Simplified Acute Physiology Score 3, *SUD* substance abuse disorder, *TBI* traumatic brain injury.

Data are presented as numbers with percentages in parentheses or medians with interquartile ranges in parentheses. *p < 0.05, **p < 0.01, ***p < 0.001.

In the extensive ICU treatment group fewer patients were female and younger on average at ICU admission. Surgery occurred more often in the extensive ICU treatment group, and patients in this group had higher SAPS3 and CCI. Also, IMV was more common and IMV time was longer. By definition, IMV > 24 h and CRRT were only seen in the extensive ICU treatment group. The patients in the extensive ICU treatment group had a longer ICU length of stay (LoS). TBI, CVA, CA, and ECMO were more common in the extensive ICU treatment group, whereas CBP was less common in the extensive vs. the non-extensive ICU treatment group. Finally, 736 (5.6%) patients in the extensive ICU treatment group suffered from incident mental disorders 1 year after ICU admission and thereafter, a numerically larger proportion than in the non-extensive ICU-treatment group.

2685 (5.3%) patients had an ICD-10 code corresponding for incident mental disorders. Stratified after Incidental mental disorders or not, patients with incident mental disorders were younger at ICU-admission, had less surgical cases, underwent less mechanical ventilation, had less time under mechanical ventilation, a lesser quota with CVA and CPB, but a larger quota with TBI. A larger proportion of patients with mental disorders received extensive ICU-treatment (Table 2).

**Table 2:** Characteristics of study patients, stratified by incident mental disorders or not.

	Mental disorders	No mental disorders	All patients
Number of patients	2685 (5.3)	48,268 (94.7)	50,953
Female sex	1162 (43.3)	20,069 (41.6)	21,231 (41.7)
Age at ICU admission (Years)***	61 (45–70)	66 (52–75)	66 (52–74)
Surgery***	267 (9.9)	6052 (12.5)	6319 (12.4)
SAPS3	51 (42–61)	52 (42–62)	52 (42–62)
APACHE II	16 (11–23.5)	16 (11–21)	16 (11–22)
CCI	0 (0–2)	0 (0–2)	0 (0–2)
IMV*	1072 (39.9)	20,494 (42.5)	21,566 (42.3)
IMV (> 24 h)*	694 (25.8)	11,514 (23.9)	12,208 (24)
IV time (minutes)***	2948 (970–8792)	2070 (500–7070)	2115 (525–7170)
CRRT	149 (5.5)	2455 (5.1)	2604 (5.1)
CRRT time (minutes)	5383 (2535–10,775)	5475 (2700–11,231)	5464.5 (2700–11,187)
IV (> 24 h) and CRRT	107 (4)	1634 (3.4)	1750 (3.4)
ICU LoS (hours)	57 (35–124)	58 (37–118)	58 (37–118)
TBI***	318 (11.8)	4169 (8.6)	4487 (8.8)
CVA***	338 (12.6)	7412 (15.4)	7750 (15.2)
CA	108 (4)	2142 (4.4)	2250 (4.4)
CPB***	215 (8)	6889 (14.3)	7014 (13.9)
ECMO	215 (8)	6889 (14.3)	7104 (13.9)
Extensive ICU treatment*	736 (27.4)	12,326 (25.5)	1889 (3.4)

*CA* cardiac arrest, *CVA* cerebrovascular accident, *CCI* Charlson comorbidity index, *CRRT* continuous renal replacement therapy, *ECMO* extracorporeal membrane oxygenation, *ICU* intensive care unit, *ISH* intentional self-harm, *IV* invasive ventilation, *LoS* length of stay, *SAPS3* Simplified Acute Physiology Score 3, *SUD* substance abuse disorder, *TBI* traumatic brain Injury.

**Figure 2:**
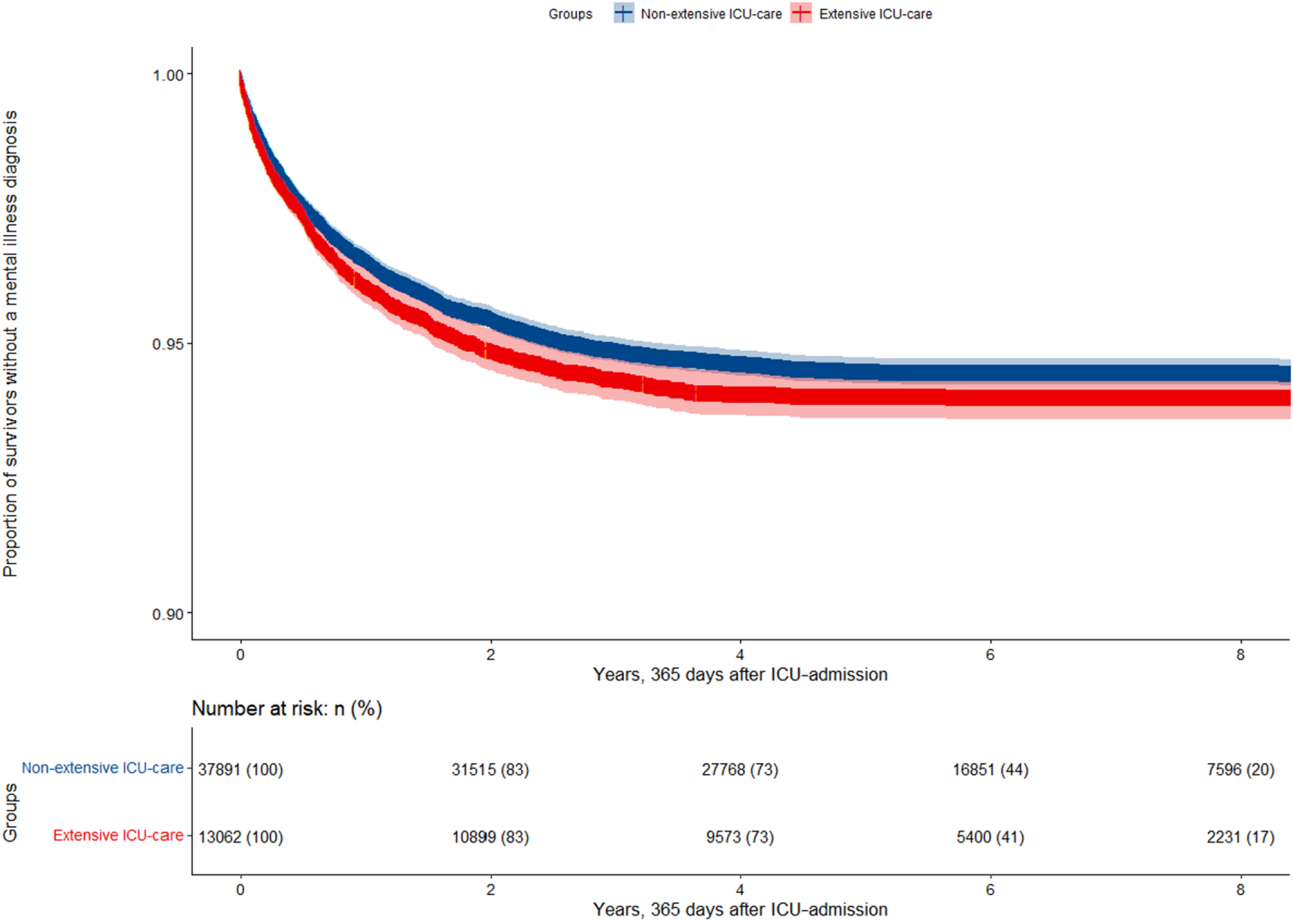
Unadjusted analysis showing survivors without mental disorders stratified after extensive and non-extensive ICU care, counting from 365 days after ICU admission, presented as Kaplan–Meier curves (95% CI). Log-rank *p* = 0.2.

Data are presented as numbers with percentages in parentheses or medians with interquartile ranges in parentheses. *p < 0.05, **p < 0.01, ***p < 0.001. Variables listed in order:

No difference was found in incident mental disorders from 365 days after ICU admission in the unadjusted comparison between the extensive and non-extensive ICU treatment groups (Fig. 2).

### Adjusted analyses

**Figure 3:**
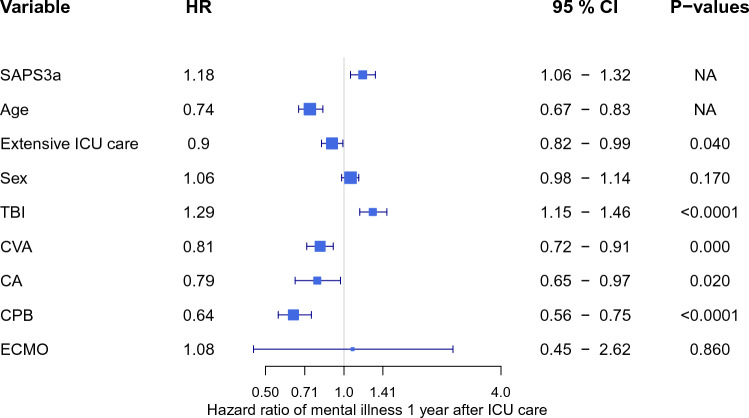
Forest-plot of Cox regression showing HRs for mental disorders > 1 year after ICU admission for patients meeting the inclusion criteria. Variables listed in order: Simplified Acute Physiology Score III (SAPS3a) adjusted for age and comorbidities; LoS length of stay, IMV invasive ventilation, CRRT continuous renal replacement therapy, TBI traumatic brain Injury, CVA cerebrovascular accident, CA cardiac arrest, SUD substance abuse disorder), ISH intentional self-harm, ECMO extracorporeal membrane oxygenation.

In the Cox model extensive ICU treatment was associated with a decreased risk of developing mental disorders (HR 0.90, 95% CI 0.82–0.99, *p* = 0.039) as were increasing age, CPB, CA, and CVA. Increasing SAPS3a and TBI was associated with an increased risk of mental disorders. Sex and ECMO did not significantly impact mental disorders (Fig. 3).

### Sensitivity analyses

The Cox model, including incident mental disorders from ICU admission and later, showed no association between extensive ICU care and increased risk of mental disorders (HR 1.02, 95% 0.95–1.09, *p* = 0.55). Performing separate models on each group of ICD-10 codes from our primary outcome (mental disorders), extensive ICU care was not associated with PTSD (HR 1.25, 95% CI 0.55–2.85, *p* = 0.60), reaction to severe stress and adjustment disorders (HR 0.98, 95% CI 0.67–1.43, *p* = 0.91), mood disorders (HR 0.95, 95% CI 0.81–1.12, *p* = 0.55), other anxiety disorders (HR 0.89, 95% CI 0.72–1.09, *p* = 0.27), or suicide (HR 1.69, 95% CI 0.49–5.78, *p* = 0.40). However, extensive ICU care was associated with decreased risk of SUD (HR 0.78, 95% CI 0.69–0.89, *p* = 0.0002) and ISH (HR 3.38, 95% CI 1.68–6.95, *p* = 0.0009). ECMO was associated with an increased risk of suicide (HR 23.74, 95% CI 1.73–326.38, *p* = 0.014). However, the number of ECMO observations was low. In the model where the extensive ICU variable was substituted with IMV > 24 h and CRRT separated, neither CRRT (HR 0.98, 95% CI 0.82–1.17, *p* = 0.83) nor IMV > 24 h (HR 0.91, 95% CI 0.83–1.01, *p* = 0.077) was associated with the risk of incident mental disorders. When we used a stricter definition of extensive ICU care requiring CRRT and IMV > 24 h, they did not correlate with mental disorders (HR 1.00, 95% CI 0.82–1.22, *p* = 1.00). Additionally, when the model was performed on complete cases only, comparable results to our main model were demonstrated, with extensive ICU care associated with a lower risk of developing mental disorders (HR 0.89, 95% CI 0.80–0.98, *p* = 0.025) and SAPS3a being significantly correlated with an increased risk of mental disorders (HR 1.20, 95% CI 1.08–1.33, *p* < 0.0001). Similarly, in the model without the TBI, CVA, CA, CPB, and ECMO variables, extensive ICU had a negative association with mental disorders (HR 0.90, 95% CI 0.82–0.99, *p* = 0.03). Finally, in the Fine-Gray analysis with death as a competing event to incident mental disorders we found no significant association with mental disorders for extensive ICU care (HR 0.94, 95% CI 0.85–1.04, *p* = 0.22).

## Discussion

ICU care is mentally challenging for patients exposed to severe illness and disturbing and often painful treatments and interventions. Contrary to our thesis, we found that treatment with mechanical ventilation, renal replacement therapy (RRT), or both, proxies for greater exposure to intensive care treatments, were associated with a decreased risk of long-term mental disorders according to our definition. This finding contrasts with previous reports in which mechanical ventilation was associated with a 10% increase in mental disorders, whereas other interventions (e.g., CRRT) were not^12^.

However, individuals with a high SAPS3a typically require extensive ICU treatment and, if not adjusted for, might bias extensive ICU care to appear as a trigger for later mental disorders. By adjusting for SAPS3a, we could separate these two factors. This adjustment might also explain why we observed a lower HR with extensive ICU care. If the underlying trigger for mental disorders is the degree of somatic illness, a higher level of treatment in the ICU might limit the risk of later mental disorders. Another potential reason for the protective effect of extensive ICU treatment might be earlier death. Because our Fine-Gray analysis with death as a competing event did not yield a significant association between extensive ICU treatment and incident mental disorders, the lower HR might be that the patients getting extensive care died before receiving a mental disorders diagnosis. The association with mental illness with SAPS3a and the negative association with extensive ICU treatment in our Cox model suggest that critical illness is the driver for developing mental disorders, not the level of care or the specific therapies.

Moreover, another reason our results deviate from some earlier findings might be that we compared different levels of ICU treatment rather than the more common contrast of ICU patients to hospitalized patients with much less illness severity, not allowing assessment of the effects of more extensive intensive care. It is also plausible that the relatively small impact of the intensity of ICU treatments is overlooked in smaller cohorts than ours. Also, the conflicting results might be related to our cohort's prevalence and incidence of mental disorders. We excluded 33 066 patients (10.5%) because they had an ICD-10 code compatible with our definition of mental disorders at inclusion. In the final cohort 5.3% (n = 2 685) of the patients were diagnosed with incident mental disorder > 1 year after ICU care. This percentage can be compared to 14–17% in other studies on large cohorts reporting mental disorders from ICU admission. Some studies, based on self-reports, disclosed depression rates of up to 34% after 12 months^6,26^. However, self-reported illness data are not comparable with clinical diagnoses because they describe the patient’s perceived symptoms rather than clinically validated mental disorders^27^.

Conversely, studies based on diagnostic codes, such as ours, have high specificity but lower sensitivity in that patients need to contact health care, get correctly diagnosed, and be diagnostically coded in the registry to be included^28^. We defined mental disorders with diagnose groups we consider being triggered by severe mental strain, thereby more specific than other epidemiological studies, including a broader spectrum of diagnoses^12,13^. We did not include diagnoses that tend to appear early in life, such as attention deficit hyperactivity disorder or personality disorders. Furthermore, we omitted ICU delirium, which has a rapid onset and is transient, thus not a long-standing mental disorder^29^. Schizophrenia was also excluded. Studies have linked childhood and early trauma to later schizophrenia^30^, however these risk factors not applicable in our study since patients < 18 years old were excluded and data on adulthood trauma as a risk factor for the development of schizophrenia are scarce^31^.

Moreover, data support that while drugs given during ICU care, such as sedatives and opiates, may modify the clinical expression and course of schizophrenia, it does not cause schizophrenia and should be viewed as drug-induced psychosis^32^. However, SUD, ISH, and suicide were included in our study because they are conditions closely linked to mental disorders or often an expression of mental disorders^33–35^.

We did not include incident mental disorders during the first year after ICU admission, ignoring short-term stress reactions, which avoided lowering specificity for long-term mental disorders. Notably, in the sensitivity analysis, where we included incident mental disorders from the date of ICU admission, no association was detected between extensive ICU care and mental disorders. We interpret this as mainly an increase in short-term anxiety and other mental disorders regardless of the level of intensive care and not lasting severe mental disorders. Moreover, it could represent identifying previous mental disorders linked to inpatient care, i.e., detection bias.

We also found that increasing age was associated with decreased risk of later mental disorders. While some studies have reported similar results^14^, others have not^12^. Generally, incident mental disorders are less likely to present from the sixth decade, which is the typical age in our cohort (36). Many patients predisposed to mental disorders may have been excluded from our study because the condition preexisted at ICU admission.

Of note, many of the conditions previously regarded as risk factors for mental disorders, including CA, CVA, and CPB, were associated with a protective effect in our model. In contrast, sex and ECMO were not associated with incident mental disorders. ECMO had a low observational number, rendering the results difficult to interpret. Because women are more prone to anxiety and mood disorders and men to SUD and suicide^36,37^, the inclusions of psychiatric disorders, which are both female- and male-dominated, possibly attenuated the sex effect in the model.

Additionally, in our study ECMO was only related to an increased risk of suicide, not an increased risk of mental disorders, differing from a recent report^13^. However, as the number of ECMO cases was low, our study is likely underpowered to detect such an effect.

### Strengths and limitations

A major strength of this study is the ability to differentiate severe illness from ICU treatments for the first time in a large cohort. Access to relevant confounding variables allowed us to model the independent effect of the extent of ICU care. Another asset is the nationwide cohort with excellent coverage and a long follow-up period (median 2020 days). The cohort included almost all ICU admissions in Sweden between 2009 and 2015, rendering a cohort large enough to detect small effect sizes. Additionally, access to ICD-10 codes for all in-hospital visits before and after ICU admission allowed us to evaluate the diagnoses of mental disorders directly instead of relying on self-reporting.

A major limitation is that we could not access primary or outpatient specialized care data. A large proportion of less severe mental disorders are treated in primary care^38^, leading to a lower sensitivity of mental disorders in this study. Yet, at the cost of losing some sensitivity, the ICD-10 codes for mental disorders in this study were registered along with inpatient care. Thus, these mental disorders are possibly more severe, long-lasting, and highly clinically significant, which are the type of mental disorders we cover in this study. Another limitation is that ICU-induced delirium, a possible confounder, is under-reported in the SIR and was therefore not included in the models. Another limitation is the rate of missing SAPS3a parameters in our study. With a data loss of 14%, the imputation rate of SAPS3a is relatively high. However, in our sensitivity analysis on only complete cases, omitting imputed data did not change the impact of extensive ICU care on mental disorders, indicating a robust imputation.

### Further research

Given that this study does not suggest severe long-standing mental disorders after extensive intensive care, a refined approach for future studies could be to add outpatient drug prescriptions as a surrogate for mental disorders and primary care data to increase sensitivity in studies of mental disorders.

## Conclusions

In a nationwide cohort of ICU patients treatment with mechanical ventilation, RRT, or both (i.e., greater exposure to intensive care treatment) was associated with a reduced risk of long-term mental disorders. However, lower age and higher illness severity was associated with an elevated risk of long-term mental disorders.

### Supplementary Information

Supplementary Information.

## Data Availability

The data used in this study are available from the SIR, the NPR, and the CDR. The data were used under license for the current study and thus not publicly available. The data, however, are available from the registries upon reasonable request after ethical review and with permission from the SIR and the Swedish National Board of Health and Welfare. Contact can be made through the corresponding author.

## Abbreviations

APACHE II: Chronic health evaluation II
CA: Cardiac arrest
CCI: Charlson comorbidity index
CVA: Cerebrovascular accident
CDR: Cause of death register
CPB: Cardiopulmonary bypass
CRRT: Continuous renal replacement therapy
ECMO: Extracorporeal membrane oxygenation
HR: Hazard ratio
ICD 10: International Classification of Diseases; Tenth Revision
ICU: Intensive care unit
ISH: Intentional self-harm
IMV: Invasive mechanical ventilation
LoS: Length of stay
NPR: National patient register
PTSD: Post-traumatic stress disorder
RRT: Renal replacement therapy
SUD: Mental and behavioral disorders due to psychoactive substance use
SAPS3: Simplified Acute Physiology Score 3
SAPS3a: Simplified Acute Physiology Score 3 adjusted for age and comorbidities
SIR: Swedish intensive care registry
TBI: Traumatic brain Injury
