# Supplementary material for: A nationwide cohort study on the association between intensive care treatments and mental distress linked psychiatric disorders
Source: Sci Rep. 2024 Feb 24;14:4519. doi: 10.1038/s41598-024-55102-9 (PMC10894289; doi:10.1038/s41598-024-55102-9)
Supplement: Supplementary file 1 — Supplementary Information. [file 41598_2024_55102_MOESM1_ESM.docx]

## A nationwide cohort study on the association between intensive care treatments and mental distress linked psychiatric disorders

Supplemantary Information

Rasmus Mossberg, MD, MSc

Björn Ahlström, MD, MSc

Miklos Lipcsey, MD, PhD

**Table S1:** Adjusted parameters

**Table S2:** ICD 10-codes for Mental disorders

**Table S3:** ICD 10-codes for other diagnoses in analysis

**Table S4:** Sensitivity analysis with the outcome PTSD instead of Mental disorders

**Table S5:** Sensitivity analysis with the outcome Reaction to severe stress and adjustment disorders instead of Mental disorders

**Table S6:** Sensitivity analysis with the outcome Mood (affective) Disorders instead of Mental disorders

**Table S7:** Sensitivity analysis with the outcome Other Anxiety Disorders instead of Mental disorders

**Table S8:** Sensitivity analysis with the outcome Intentional Self Harm instead of Mental disorders

**Table S9:** Sensitivity analysis with the outcome Mental and behavioral disorders due to psychoactive substance use instead of Mental disorders

**Table S10:** Sensitivity analysis with the outcome suicide instead of Mental disorders

**Table S11:** Sensitivity analysis against IV 24 hours and CRRT instead of extensive ICU care

**Table S12:** analysis against CRRT and IV>24h separate instead of Extensive ICU care

**Table S13:** Sensitivity analysis without controlling for diagnoses known to affect Mental disorders

**Table S14:** Sensitivity analysis including incident Mental disorders from ICU admission

**Table S15:** Only complete cases

**Table S16:** Fine-Gray analysis

**Table S1:** Adjusted parameters

| **Adjusted parameter** | **Formula** |
| --- | --- |
| SAPS3a | SAPS3 – ([points for age] + [points for comorbidities]) |
| Extensive ICU care | ([IV>24 hours] AND/OR [CRRT]) |
| IV 24 hours and CRRT | ([IV>24 hours] AND [CRRT]) |

**Table S2:** ICD 10-codes for the outcome, Mental disorders

| **Diagnosis** | **ICD 10-codes** |
| --- | --- |
| Mental disorders | Any or one of the following: |
| Mood (affective) Disorders | F30-F39 |
| Reaction to severe stress and adjustment disorders | F43 |
| Post-traumatic stress disorder | F43.1 |
| Suicide | X60-X69, X70-X79, X80-X84, Y10-Y19, Y20-29, Y30-34 (as reason of death) |
| Mental and behavioral disorders due to psychoactive substance use | F10-F19 |
| Intentional Self Harm | X60-X69, X70-X79, X80-X84 |
| Other Anxiety Disorders | F41 |

**Table S3:** ICD 10-codes for other diagnoses in analysis

| **Diagnosis** | **ICD 10-codes/Operational codes** |
| --- | --- |
| Traumatic Brain Injury | S00-S09 |
| Cerebrovascular accident | G45, I63, I64, I60, I61, I62.0, I162.1, I162.9 (1) |
| Cardiac Arrest | I460, I461, I469, I4900, I4901, R960, R961, R98, R99 (2) |
| Cardiopulmonary bypass | FXA00, FXA10, FXA13, FXA96, FXB00, FXC00, FXL10, FXL20, FXL30, FXM10, FXM20, FXM30, FXN00 |
| Extracorporeal Membrane Oxygenation | DV023, DXD00 |

**Table S4:** Sensitivity analysis with the outcome PTSD instead of Mental disorders

| **Parameters** | **HR** | **95% CI** | **P-value** |
| --- | --- | --- | --- |
| SAPS3a | 0.66 | 0.22-1.94 | 0.19 |
| Age | 0.05 | 0.01-0.3 | 0.036 |
| Extensive ICU care | 1.25 | 0.55-2.85 | 0.60 |
| Gender | 1.15 | 0.58-2.27 | 0.27 |
| Traumatic Brain Injury | 0.45 | 0.11-1.89 | 0.37 |
| Cerebrovascular accident | 0.52 | 0.12-2.19 | 0.97 |
| Cardiac Arrest | 1.04 | 0.13-8.28 | 0.61 |
| Cardiopulmonary bypass | 0 | 0-1.59E+7 | 0.96 |
| Extracorporeal Membrane Oxygenation | 0.02 | 0-1.22E+62 | 0.27 |

**Table S5:** Sensitivity analysis with the outcome Reaction to severe stress and adjustment disorders instead of Mental disorders

| **Parameters** | **HR** | **95% CI** | **P-value** |
| --- | --- | --- | --- |
| SAPS3a | 0.72 | 0.46-1.11 | 0.43 |
| Age | 0.18 | 0.11-0.28 | 0.0009 |
| Extensive ICU care | 0.98 | 0.67-1.43 | 0.91 |
| Gender female | 1.49 | 1.1-2 | 0.0091 |
| Traumatic Brain Injury | 0.71 | 0.42-1.21 | 0.21 |
| Cerebrovascular accident | 1.21 | 0.78-1.89 | 0.39 |
| Cardiac Arrest | 1.53 | 0.74-3.16 | 0.25 |
| Cardiopulmonary bypass | 0.37 | 0.16-0.86 | 0.021 |
| Extracorporeal Membrane Oxygenation | 0.06 | 0-3.78E+5 | 0.73 |

**Table S6:** Sensitivity analysis with the outcome Mood (affective) Disorders instead of Mental disorders

| **Parameters** | **HR** | **95% CI** | **P-value** |
| --- | --- | --- | --- |
| SAPS3a | 0.95 | 0.78-1.16 | 0.0002 |
| Age | 0.74 | 0.62-0.89 | 0.19 |
| Extensive ICU care | 0.95 | 0.81-1.12 | 0.55 |
| Female gender | 1.56 | 1.37-1.78 | <.0001 |
| Traumatic Brain Injury | 1.22 | 0.99-1.51 | 0.06 |
| Cerebrovascular accident | 0.89 | 0.73-1.07 | 0.21 |
| Cardiac Arrest | 0.71 | 0.49-1.03 | 0.069 |
| Cardiopulmonary bypass | 0.85 | 0.67-1.07 | 0.16 |
| Extracorporeal Membrane Oxygenation | 1.75 | 0.56-5.48 | 0.34 |

**Table S7:** Sensitivity analysis with the outcome Other Anxiety Disorders instead of Mental disorders

| **Parameters** | **HR** | **95% CI** | **P-value** |
| --- | --- | --- | --- |
| SAPS3a | 1.29 | 1.01-1.65 | 0.0025 |
| Age | 0.74 | 0.59-0.94 | 0.94 |
| Extensive ICU care | 0.89 | 0.72-1.09 | 0.27 |
| Female gender | 1.7 | 1.44-2.01 | <.0001 |
| Traumatic Brain Injury | 0.89 | 0.66-1.21 | 0.46 |
| Cerebrovascular accident | 0.75 | 0.58-0.98 | 0.034 |
| Cardiac Arrest | 0.86 | 0.55-1.34 | 0.50 |
| Cardiopulmonary bypass | 0.6 | 0.42-0.85 | 0.0038 |
| Extracorporeal Membrane Oxygenation | 0.05 | 0-337 | 0.51 |

**Table S8:** Sensitivity analysis with the outcome Intentional Self Harm instead of Mental disorders

| **Parameters** | **HR** | **95% CI** | **P-value** |
| --- | --- | --- | --- |
| SAPS3a | 0.89 | 0.36-2.2 | 0.91 |
| Age | 0.13 | 0.04-0.39 | 0.011 |
| Extensive ICU care | 3.38 | 1.64-6.96 | 0.0009 |
| Female gender | 1.2 | 0.62-2.32 | 0.60 |
| Traumatic Brain Injury | 1.88 | 0.84-4.23 | 0.13 |
| Cerebrovascular accident | 0.36 | 0.08-1.5 | 0.16 |
| Cardiac Arrest | 0.4 | 0.05-3.08 | 0.38 |
| Cardiopulmonary bypass | 0 | 0-1.02E+7 | 0.61 |
| Extracorporeal Membrane Oxygenation | 0.01 | 0-1.6952E+60 | 0.95 |

**Table S9:** Sensitivity analysis with the outcome Mental and behavioral disorders due to psychoactive substance use instead of Mental disorders

| **Parameters** | **HR** | **95% CI** | **P-value** |
| --- | --- | --- | --- |
| SAPS3a | 1.39 | 1.2-1.62 | <.0001 |
| Age | 0.61 | 0.53-0.7 | 0.0002 |
| Extensive ICU care | 0.79 | 0.69-0.89 | <.0001 |
| Female gender | 0.72 | 0.64-0.8 | <.0001 |
| Traumatic Brain Injury | 1.5 | 1.29-1.74 | <.0001 |
| Cerebrovascular accident | 0.71 | 0.6-0.83 | <.0001 |
| Cardiac Arrest | 0.79 | 0.61-1.03 | 0.086 |
| Cardiopulmonary bypass | 0.54 | 0.43-0.67 | <.0001 |
| Extracorporeal Membrane Oxygenation | 1.18 | 0.38-3.72 | 0.77 |

**Table S10:** Sensitivity analysis with the outcome suicide instead of Mental disorders

| **Parameters** | **HR** | **95% CI** | **P-value** |
| --- | --- | --- | --- |
| SAPS3a | 0.37 | 0.07-1.98 | 0.25 |
| Age | 0.29 | 0.04-1.89 | 0.78 |
| Extensive ICU care | 1.69 | 0.49-5.78 | 0.40 |
| Female gender | 0.19 | 0.04-0.86 | 0.030 |
| Traumatic Brain Injury | 0 | 0-1.57E+16 | 0.75 |
| Cerebrovascular accident | 0 | 0-8.78E+13 | 0.74 |
| Cardiac Arrest | 0.61 | 0.06-6.32 | 0.68 |
| Cardiopulmonary bypass | 0.34 | 0.03-3.41 | 0.36 |
| Extracorporeal Membrane Oxygenation | 23.74 | 1.73-326.38 | 0.018 |

**Table S11:** Sensitivity analysis against IV 24 hours and CRRT instead of extensive ICU care

| **Parameters** | **HR** | **95% CI** | **P-value** |
| --- | --- | --- | --- |
| SAPS3a | 1.15 | 1.03-1.28 | 0.0003 |
| IV 24 hours and CRRT | 1 | 0.82-1.22 | 1.00 |
| Age | 0.75 | 0.67-0.83 | <.0001 |
| Female gender | 1.06 | 0.98-1.14 | 0.15 |
| Traumatic Brain Injury | 1.28 | 1.14-1.45 | <.0001 |
| Cerebrovascular accident | 0.81 | 0.72-0.91 | 0.0003 |
| Cardiac Arrest | 0.78 | 0.64-0.95 | 0.014 |
| Cardiopulmonary bypass | 0.65 | 0.56-0.75 | <.0001 |
| Extracorporeal Membrane Oxygenation | 1.05 | 0.43-2.57 | 0.91 |

**Table S12:** analysis against CRRT and IV>24h separate instead of Extensive ICU care

| **Parameters** | **HR** | **95% CI** | **P-value** |
| --- | --- | --- | --- |
| SAPS3a | 1.18 | 1.06-1.32 | 0.0005 |
| IV >24 hours | 0.92 | 0.83-1.01 | 0.077 |
| CRRT | 0.98 | 0.82-1.17 | 0.83 |
| Age | 0.74 | 0.67-0.83 | <.0001 |
| Female gender | 1.06 | 0.98-1.14 | 0.17 |
| Traumatic Brain Injury | 1.29 | 1.15-1.46 | <.0001 |
| Cerebrovascular accident | 0.81 | 0.72-0.91 | 0.0004 |
| Cardiac Arrest | 0.79 | 0.65-0.97 | 0.025 |
| Cardiopulmonary bypass | 0.64 | 0.56-0.75 | <.0001 |
| Extracorporeal Membrane Oxygenation | 1.09 | 0.45-2.64 | 0.85 |

**Table S13:** Sensitivity analysis without controlling for diagnoses known to affect Mental disorders

| **Parameters** | **HR** | **95% CI** | **P-value** |
| --- | --- | --- | --- |
| SAPS3a | 1.22 | 1.09-1.36 | 0.0003 |
| Age | 0.71 | 0.64-0.79 | <.0001 |
| Extensive ICU care | 0.9 | 0.82-0.99 | 0.034 |
| Female gender | 1.07 | 0.99-1.15 | 0.092 |

**Table S14:** Sensitivity analysis including incident Mental disorders from ICU admission

| **Parameters** | **HR** | **95% CI** | **P-value** |
| --- | --- | --- | --- |
| SAPS3a | 1.08 | 1-1.17 | <.0001 |
| Age | 0.64 | 0.59-0.69 | <.0001 |
| Extensive ICU care | 1.02 | 0.95-1.09 | 0.55 |
| Female gender | 1.03 | 0.97-1.09 | 0.29 |
| Traumatic Brain Injury | 1.21 | 1.05-1.39 | 0.0066 |
| Cerebrovascular accident | 0.83 | 0.71-0.97 | 0.017 |
| Cardiac Arrest | 0.95 | 0.77-1.16 | 0.59 |
| Cardiopulmonary bypass | 0.6 | 0.54-0.67 | <.0001 |
| Extracorporeal Membrane Oxygenation | 1.52 | 0.87-2.63 | 0.14 |

**Table S15:** Only complete cases

| **Parameters** | **HR** | **95% CI** | **P-value** |
| --- | --- | --- | --- |
| SAPS3a | 1.2 | 1.08-1.33 | <.0001 |
| Age | 0.75 | 0.67-0.84 | <.0001 |
| Extensive ICU care | 0.89 | 0.81-0.99 | 0.025 |
| Female gender | 1.06 | 0.98-1.15 | 0.15 |
| Traumatic Brain Injury | 1.28 | 1.13-1.45 | 0.0001 |
| Cerebrovascular accident | 0.78 | 0.69-0.89 | 0.0001 |
| Cardiac Arrest | 0.82 | 0.67-1.01 | 0.065 |
| Cardiopulmonary bypass | 0.75 | 0.61-0.91 | 0.0042 |
| Extracorporeal Membrane Oxygenation | 1.22 | 0.5-2.98 | 0.66 |

**Table S16:** Fine-Gray analysis

| **Parameters** | **HR** | **95% CI** | **P-value** |
| --- | --- | --- | --- |
| SAPS3a | 1.05 | 1.03-1.07 | <.0001 |
| SAPS3a’ | 0.88 | 0.81-0.95 | 0.001 |
| SAPS3a’’ | 1.34 | 1.11-1.61 | 0.0025 |
| Extensive ICU care | 0.94 | 0.86-1.04 | 0.22 |
| Age | 0.99 | 0.99-0.99 | <.0001 |
| Female gender | 0.96 | 0.89-1.04 | 0.33 |
| Traumatic Brain Injury | 1.27 | 1.13-1.43 | <.0001 |
| Cerebrovascular accident | 0.82 | 0.73-0.92 | 0.0009 |
| Cardiac Arrest | 0.82 | 0.67-1 | 0.047 |
| Cardiopulmonary bypass | 0.68 | 0.59-0.79 | <.0001 |
| Extracorporeal Membrane Oxygenation | 1.07 | 0.44-2.59 | 0.88 |

CRRT: Continuous renal replacement therapy

ICD 10: International Classification of Diseases 10th Revision

ICU: Intensive Care Unit

IV: Invasive Ventilation

PTSD: Post-traumatic stress disorder

SAPS3: Simplified Acute Physiology Score III

SAPS3a: Simplified Acute Physiology Score III adjusted

1. Antoniou T, Macdonald EM, Yao Z, et al (2017) Association between statin use and ischemic stroke or major hemorrhage in patients taking dabigatran for atrial fibrillation. Cmaj 189:E4–E10 . doi: 10.1503/cmaj.160303

2. Wong MKY, Morrison LJ, Qiu F, et al (2014) Trends in Short- and Long-Term Survival Among Out-of-Hospital Cardiac Arrest Patients Alive at Hospital Arrival. Circulation. doi: 10.1161/CIRCULATIONAHA.114.010633
